# GP60 and SPARC as albumin receptors: key targeted sites for the delivery of antitumor drugs

**DOI:** 10.3389/fphar.2024.1329636

**Published:** 2024-01-23

**Authors:** Qingzhi Ji, Huimin Zhu, Yuting Qin, Ruiya Zhang, Lei Wang, Erhao Zhang, Xiaorong Zhou, Run Meng

**Affiliations:** ^1^ School of Pharmacy, Yancheng Teachers University, Yancheng, China; ^2^ Sheyang County Comprehensive Inspection and Testing Center, Yancheng, China; ^3^ Department of Immunology, Medical School, Nantong University, Nantong, China

**Keywords:** GP60, SPARC, albumin, target delivery, drug carriers

## Abstract

Albumin is derived from human or animal blood, and its ability to bind to a large number of endogenous or exogenous biomolecules makes it an ideal drug carrier. As a result, albumin-based drug delivery systems are increasingly being studied. With these in mind, detailed studies of the transport mechanism of albumin-based drug carriers are particularly important. As albumin receptors, glycoprotein 60 (GP60) and secreted protein acidic and rich in cysteine (SPARC) play a crucial role in the delivery of albumin-based drug carriers. GP60 is expressed on vascular endothelial cells and enables albumin to cross the vascular endothelial cell layer, and SPARC is overexpressed in many types of tumor cells, while it is minimally expressed in normal tissue cells. Thus, this review supplements existing articles by detailing the research history and specific biological functions of GP60 or SPARC and research advances in the delivery of antitumor drugs using albumin as a carrier. Meanwhile, the deficiencies and future perspectives in the study of the interaction of albumin with GP60 and SPARC are also pointed out.

## 1 Introduction

Albumin is derived from blood plasma and is the most abundant protein in the blood, accounting for approximately 50% of the total protein in the plasma (Forsthuber et al., 2020; Jagdish et al., 2021). The common types of albumin include human serum albumin (HSA), bovine serum albumin (BSA), equine serum albumin (ESA), and murine serum albumin (MSA). Although there are many types of albumin, HSA and BSA are the most common albumin types used in biomedical research works and applications. The crystal structures of the proteins of HSA and BSA are similar, and both show a heart-shaped structure. HSA consists of 585 amino acids, and the molecular weight of HSA is approximately 69.367 kDa. BSA consists of 583 amino acids, and the molecular weight of BSA is approximately 69.293 kDa. The isoelectric points of both native HSA and BSA are between 4.7 and 4.9. Because albumin has the advantages of non-immunogenicity, non-cytotoxicity, and multiple drug-binding sites, it has a wide range of applications in the biomedical field (Hu et al., 2022), especially in drug delivery systems. There are many albumin drug-binding sites in the human body, such as GP60, GP30, GP18, secreted protein acidic and rich in cysteine (SPARC), FcRn, cubilin, and megalin (Molitoris et al., 2022). Among these albumin receptors, GP60 and SPARC play key roles in the delivery of antitumor drugs based on albumin drug carriers.

To date, there are some reviews that summarize advances in albumin-based drug delivery. However, most of these reviews focus more on describing the preparation methods of albumin nanoparticles, drug-loading types, and the current research progress of albumin nanoparticles, and few reviews detailing GP60 and SPARC are available (Spada et al., 2021; Ishima et al., 2022; Paul et al., 2022). As a supplement, this review details the research history, biological functions, and current research advances of GP60 and SPARC and the existing problems and future perspectives about albumin-based drug carriers constructed by the GP60- and SPARC-mediated pathway.

## 2 The research history of GP60 and SPARC

**FIGURE 1:**
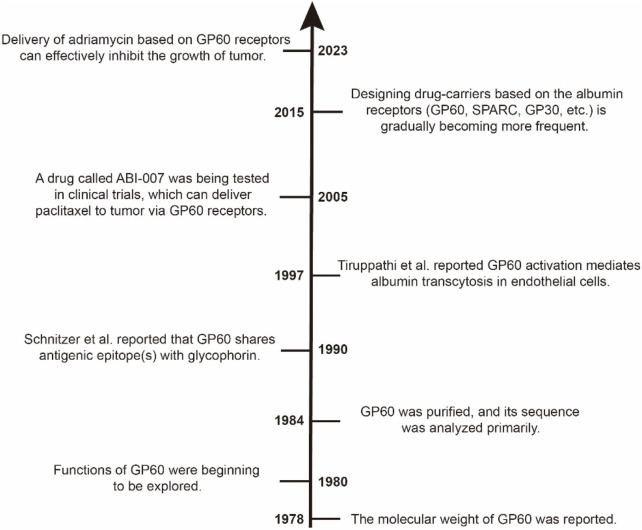
Timeline of the research about GP60 receptors. In the 1980s, GP60 was purified and characterized. In the 1990s, the functions of GP60 were reported one after another. Since the beginning of the 21st century, albumin–GP60 interaction and albumin–SPARC interaction have been successively applied to studies of drug delivery, especially in the last decade.

GP60, also called albondin, is a 60-kDa microvascular endothelial glycoprotein. In 1978, Rohde et al. described a kind of glycoprotein whose molecular weight is 60 kDa (Rohde et al., 1978). Yagi et al. explored the functions of GP60 based on a mouse mammary tumor in 1980, and they found that the presence of GP60 in mature B-type virions is related to the environment of the MJY-alpha cells (Yagi et al., 1980). Then, in 1984, GP60 was purified by Schultz et al. from bovine leukemia virus (BLV) using controlled pore glass and reverse-phase liquid chromatography (RPLC), and they also analyzed the amino acid sequence of the purified GP60 (Schultz et al., 1984). Around the 1990s, Schnitzer et al. found that GP60 is expressed on vascular endothelial cells and that it can interact with albumin to allow albumin to be transported across the endothelium (Schnitzer et al., 1988; Schnitzer, 1992). Milici and Tiruppathi also found that GP60 not only binds natural albumin but also facilitates its internalization and transcytosis (Milici et al., 1987; Tiruppathi et al., 1997). It has been shown that approximately 50% of albumin crossing the vascular endothelium is dependent on the GP60 receptor, and the rest of albumin crosses this barrier mainly through intercellular junctions and the fluid-phase mechanism (Schnitzer, 1993; Schnitzer and Oh, 1994). Iancu, Schnitzer, and Tiruppathi found that albumin cellular internalization occurs mainly through the caveolin-dependent endocytotic process (Schnitzer et al., 1995; Tiruppathi et al., 1997; Iancu et al., 2011). In 1996, the GP60 receptor from vascular endothelial cells was isolated and characterized by Tiruppathi et al., and their findings also indicated that the GP60 receptor on the surface of endothelial cells mediates the specific binding of native albumin to endothelial cells and, thus, may regulate the uptake of albumin and its transcytosis (Tiruppathi et al., 1996). Based on the albumin–GP60 interaction, a drug called ABI-007 was constructed successfully, and it could deliver paclitaxel to the tumor through GP60 receptors on the surface of vascular endothelial cells (Nyman et al., 2005). Shortly thereafter, the famous antitumor drug named Abraxane^®^ was approved for marketing by the U.S. Food and Drug Administration. In the last decade, a lot of research studies based on the albumin–GP60 interaction started to emerge. For example, albumin-consolidated AIEgens were constructed for boosting glioma and cerebrovascular NIR-II fluorescence imaging, and these albumin-based AIE nanoprobes enable the limited fluorescence imaging-guided surgery of brain tumor and cerebral ischemia (Gao et al., 2023). Also, protein nanoparticles constructed via the albumin–GP60 interaction demonstrated a strong ability to overcome cancer drug resistance, and it was expected to be further used in clinical practice (Hassanin and Elzoghby, 2020). Cisplatin-loaded albumin–gold nanoparticles could interact with glycans of the GP60 receptor, and the mechanisms of this interaction were explored at the molecular and cellular levels by Jaiswal et al.; thus, this finding could be effectively used for *in vivo* or *in vitro* targeted drug delivery applications to cure cancer (Jaiswal et al., 2023). Kumari et al. used BSA to modify metal nanoparticles in order to make these metal nanoparticles interact with glycans of the GP60 receptor on endothelial cells for targeted drug delivery, and they also explored in detail the mechanism of interaction between albumin and glycans of the GP60 receptor; thus, these findings could form a promising platform to investigate the interaction of albumin nanoparticles with the GP60 receptor in both *in vitro* and *in vivo* applications for targeted drug delivery therapy (Kumari et al., 2022). A brief research history of the GP60 receptor is shown in Figure 1.

**FIGURE 2:**
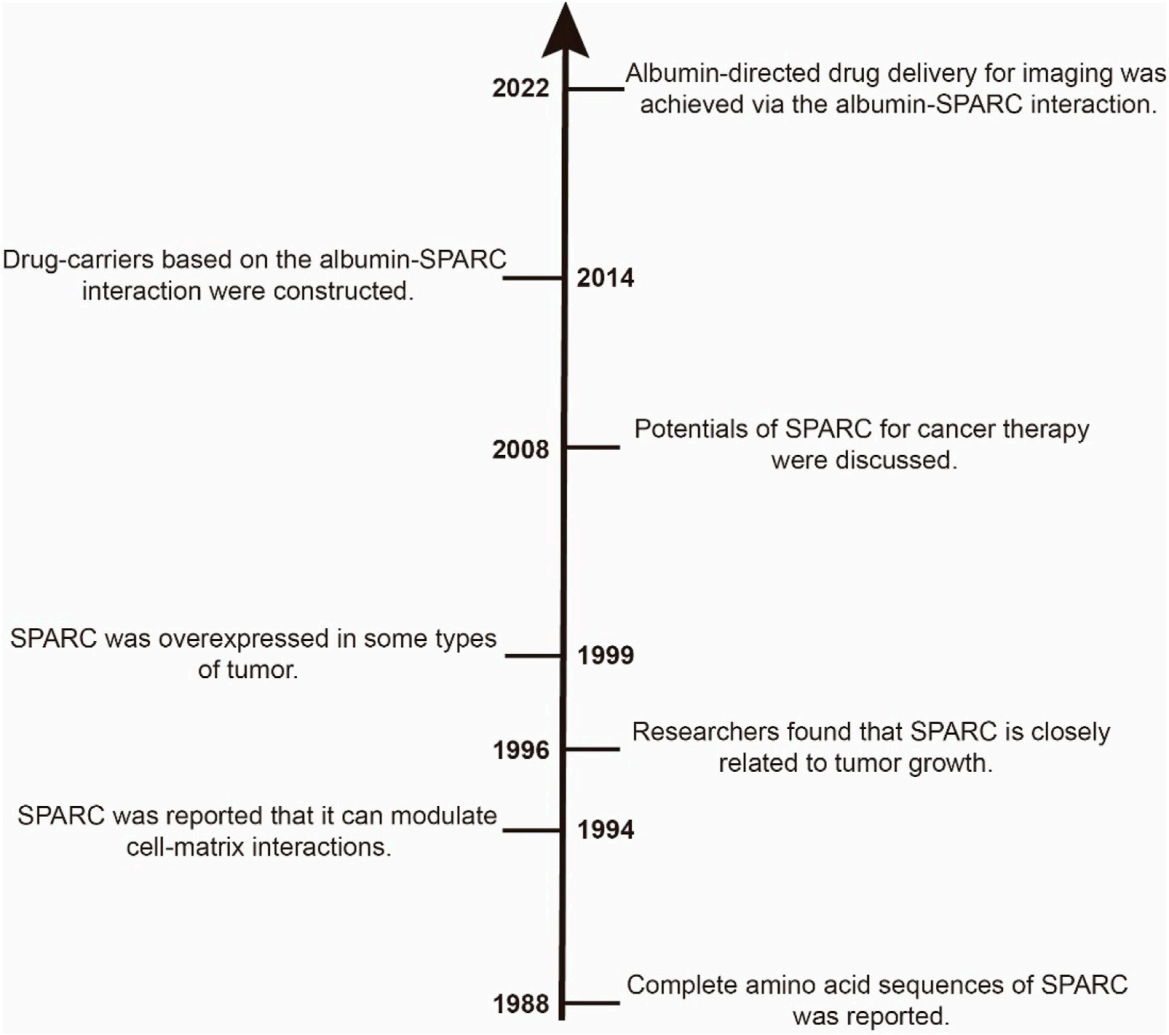
Timeline of the research about SPARC. In the 1980s, the physical and chemical characteristics of SPARC were successively identified. Then, SPARC was found to be similar to GP60 receptors, and its functions were successively reported in the 1990s. In the first decade of the 21st century, the potentials of SPARC were primarily discussed and explored. In the last decade, more and more research studies reported applications of the albumin–SPARC interaction.

SPARC is also known as osteonectin or BM-40 (basement membrane 40). SPARC is a single-copy gene that is highly conserved, with over 70% amino acid sequence homology in various sequences. The length of amino acids of SPARC derived from humans is 303, and its molecular weight is approximately 34.632 kDa (UniProt entry: P09486). Termine et al. first identified SPARC as a major non-collagenous component of bone in 1981 and found that SPARC is a cysteine-rich, low-molecular-weight glycoprotein (Termine et al., 1981). In 1988, the sequence of complete amino acids of SPARC was reported (Lankat-Buttgereit et al., 1988). Lane and Sage conducted a series of experiments about SPARC. Their results showed that SPARC could inhibit cell cycle progression *in vitro*, in part through a cationic, disulfide-bonded region. Moreover, SPARC could bind to the B chain of the platelet-derived growth factor and alter the response of cells to several cytokines (Lane and Sage, 1994). In 1996, Jendraschak et al. investigated how SPARC regulates angiogenesis. The results showed that SPARC might act pleiotropically during angiogenesis in conjunction with other known angiogenic factors (Jendraschak and Sage, 1996). Since 1999, SPARC has been consistently reported to be overexpressed in various types of tumors, such as breast cancer, liver cancer, neuroblastoma, and glioma (Le Bail et al., 1999; Gorantla et al., 2013; Lin et al., 2016; Gao et al., 2021). In 2008, a review by Tai and Tang introduced in detail the role of SPARC in cancer progression and its potential for cancer therapy, and the role of SPARC in sensitizing therapy-resistant cancer types was also discussed (Tai and Tang, 2008). Over the past decade, more and more biomedical applications focused on drug delivery systems based on the albumin–SPARC interaction have been developed. Applications of SPARC in the biomedical field include the construction of drug delivery systems and the preparation of imaging agents and diagnostic reagents (Nagaraju and El-Rayes, 2013; Hu et al., 2022; Jiang et al., 2023). A brief research history of SPARC is shown in Figure 2.

## 3 Specific biological functions of GP60 and SPARC

In this review, we focused on the function of GP60 and SPARC as albumin receptors. However, GP60 or SPARC carries out many biological functions in addition to its role as an albumin receptor. Thus, in order to provide a comprehensive understanding of GP60 and SPARC, we also provide a comprehensive overview of their other biological functions.

### 3.1 GP60

GP60 receptors are expressed in vascular endothelial cells except in brain tissues, and they enable the transport of albumin from the inside to the outside of blood vessels in 13 s (Stewart, 2000; Hama et al., 2021). It is important to note that the distribution of HSA regulated by SPARC depends on blood volume and protein status *in vivo*. Albumin first binds to the GP60 receptor, which in turn binds further to an intracellular protein called caveolin-1. Then, the cell membrane invaginates to produce transcellular vesicles, which eventually allows albumin to cross the vascular endothelium (John et al., 2001). So, specific biological functions or characteristics of GP60 as an albumin receptor include overexpression in tumor tissues and mediating albumin transcytosis in endothelial cells. These functions and features of GP60 provide a rationale for its use as a therapeutic target for tumors, as well as the theoretical basis for the design of various types of drug carriers based on the albumin–GP60 interaction. Table 1 details the known functions of GP60 and the reported years.

**TABLE 1:** Functions or characteristics of GP60 and SPARC.

Name	Functions or characteristics	References
GP60	As an albumin receptor	Schnitzer et al. (1988); Schnitzer (1992); Tiruppathi et al. (1997); Sleep (2015)
	Overexpressed in HUVEC cells	Maniatis et al. (2006); Kumari et al. (2019); Paul et al. (2022)
	Activating vesicle formation and trafficking	Minshall et al. (2000)
	GP60 is immunologically related to glycophorin	Schnitzer et al. (1990)
	Mediating albumin transcytosis in endothelial cells	Tiruppathi et al. (1997)
	Binding *Limax flavus*, *Ricinus communis*, and *Triticum vulgare* agglutinins but not other lectins	Schnitzer et al. (1990)
	GP60 is sequentially precipitated from 125I-labeled cell lysates by using *R. communis* agglutinin followed by *T. vulgare* agglutinin	Schnitzer et al. (1990)
	GP60 is sensitive to sialidase digestion	Schnitzer et al. (1990)
SPARC	Interacting with albumin	Wan et al. (2015); Ruan et al. (2018); Zhang et al. (2018)
	Overexpressed in some types of tumors, such as breast cancer, highly metastatic tumors, and human hepatocellular carcinoma	Le Bail et al. (1999); Feng and Tang (2014); Li et al. (2022)
	Regulating the cellular secretion rates of fibronectin and laminin extracellular matrix proteins	Kamihagi et al. (1994)
	Triggering a cell-autonomous program of synapse elimination	López-Murcia et al. (2015)
	Regulating collagen interaction with cardiac fibroblast cell surfaces	Harris et al. (2011)
	Interacting with AMPK and regulating GLUT4 expression	Song et al. (2010)
	Contributing to adipose tissue formation	Atorrasagasti et al. (2022)
	Increased SPARC expression promotes U87 glioblastoma invasion	Golembieski et al. (1999)
	Expressed in renal interstitial fibrosis	Pichler et al. (1996)
	Modulating the cell growth, attachment, and migration of U87 glioma cells	Rempel et al. (2001)
	Inducing inflammatory interferon response	Ryu et al. (2022)
	Promoting leukemic cell growth	Alachkar et al. (2014)
	SPARC was overexpressed in human endometrial cancer stem-like cells and promoted migration activity	Yusuf et al. (2014)
	Promoting pericyte recruitment via the inhibition of endoglin-dependent TGF-β1 activity	Rivera and Brekken (2011)
	Stimulating the neuronal differentiation of medulloblastoma cells via the Notch1/STAT3 pathway	Bhoopathi et al. (2011)
	Accelerating disease progression in experimental crescentic glomerulonephritis	Sussman et al. (2009)
	SPARC deficiency affects bone marrow stromal function	Luo et al. (2014)
	SPARC is associated with gastric cancer progression	Zhao et al. (2010)
	Promoting cathepsin B-mediated melanoma invasiveness	Girotti et al. (2011)
	Inhibiting adipogenesis by its enhancement of beta-catenin signaling	Nie and Sage (2009)
	Inducing cell cycle arrest via the STAT3 signaling pathway in medulloblastoma cells	Chetty et al. (2012)
	SPARC is upregulated during skeletal muscle regeneration and inhibits myoblast differentiation	Petersson et al. (2013)
	Mediating the src-induced disruption of the actin cytoskeleton	Bhoopathi et al. (2011)
	SPARC downregulation attenuates the profibrogenic response of hepatic stellate cells	Atorrasagasti et al. (2011)
	SPARC is a key regulator of proliferation, apoptosis, and invasion in human ovarian cancer	Chen et al. (2012)
	Overexpressed SPARC promotes liver cancer cell proliferation and tumor growth	Gao et al. (2021)
	A key mediator of TGF-β-induced renal cancer metastasis	Bao et al. (2021)
	Regulating endothelial cell shape and barrier function	Goldblum et al. (1994)
	SPARC is a Ca^2+^-binding and stress-related protein	Sage et al. (1989); Sage et al. (1989); Funk and Sage (1991)
	Regulating ferroptosis	Hua et al. (2021)

### 3.2 SPARC

The biological functions of SPARC that are now well defined include its role as a protein that interacts with albumin, regulating the cellular secretion rates of fibronectin and laminin extracellular matrix proteins, regulating cell proliferation, preventing cellular adhesion, promoting cellular deformation, regulating cell differentiation, inhibiting cellular response to some growth factors, and regulating the production of the extracellular matrix and metalloproteinase. Compared to the GP60 receptor, SPARC exhibits more biological functions *in vivo* (Rivera et al., 2011; Bradshaw, 2016; Chen et al., 2020). Here, the role of SPARC in the occurrence and development of tumors is highlighted. To date, there are at least four functions of SPARC identified as relevant to tumors. First, SPARC has an anti-adhesive effect. This function is different from most of the extracellular matrix (ECM) constituents, and the function shows some concentration dependence (Volmer et al., 2004). SPARC interferes with cell surface binding to ECM components and interacts with growth factors to change the cell shape. The separation of cells is the beginning of tumor invasion of surrounding tissues and distant metastasis, which has a key role in the progression of malignant tumors. Second, SPARC can degrade the ECM. SPARC induces the synthesis of collagenase, gelatinase, the mesenchymal degrading enzyme, etc., to degrade the matrix and impair the function of the matrix barrier, which ultimately promotes the metastasis of tumor cells. Third, SPARC is involved in the regulation of multiple signaling pathways. These pathways include the PI3K–Akt–mTOR pathway, mitogen-activated protein kinase (MAPK)/extracellular signal-regulated pathway, WNT/beta-catenin signaling pathway, and endothelial paracellular pathway via protein tyrosine phosphorylation. Finally, SPARC can modulate angiogenesis. As early as 1996, Jendraschak and Sage summarized the findings that SPARC can modulate angiogenesis. The findings showed that SPARC can promote the lysis of the basement membrane and the movement of endothelial cells. The hydrolysis product of SPARC, named calcium-binding peptide, can stimulate angiogenesis and cell growth. Therefore, in the early stages of malignant transformation, SPARC plays a crucial role in tumor cell proliferation and metastasis, angiogenesis, and the remodeling of the extracellular matrix. SPARC is overexpressed in progressively aggressive tumors, which may be indicative of a failure of homeostatic repair between the tissue and the microenvironment. More functions or characteristics of SPARC are summarized in Table 1.

## 4 Antitumor drug delivery targeting GP60 and SPARC

**FIGURE 3:**
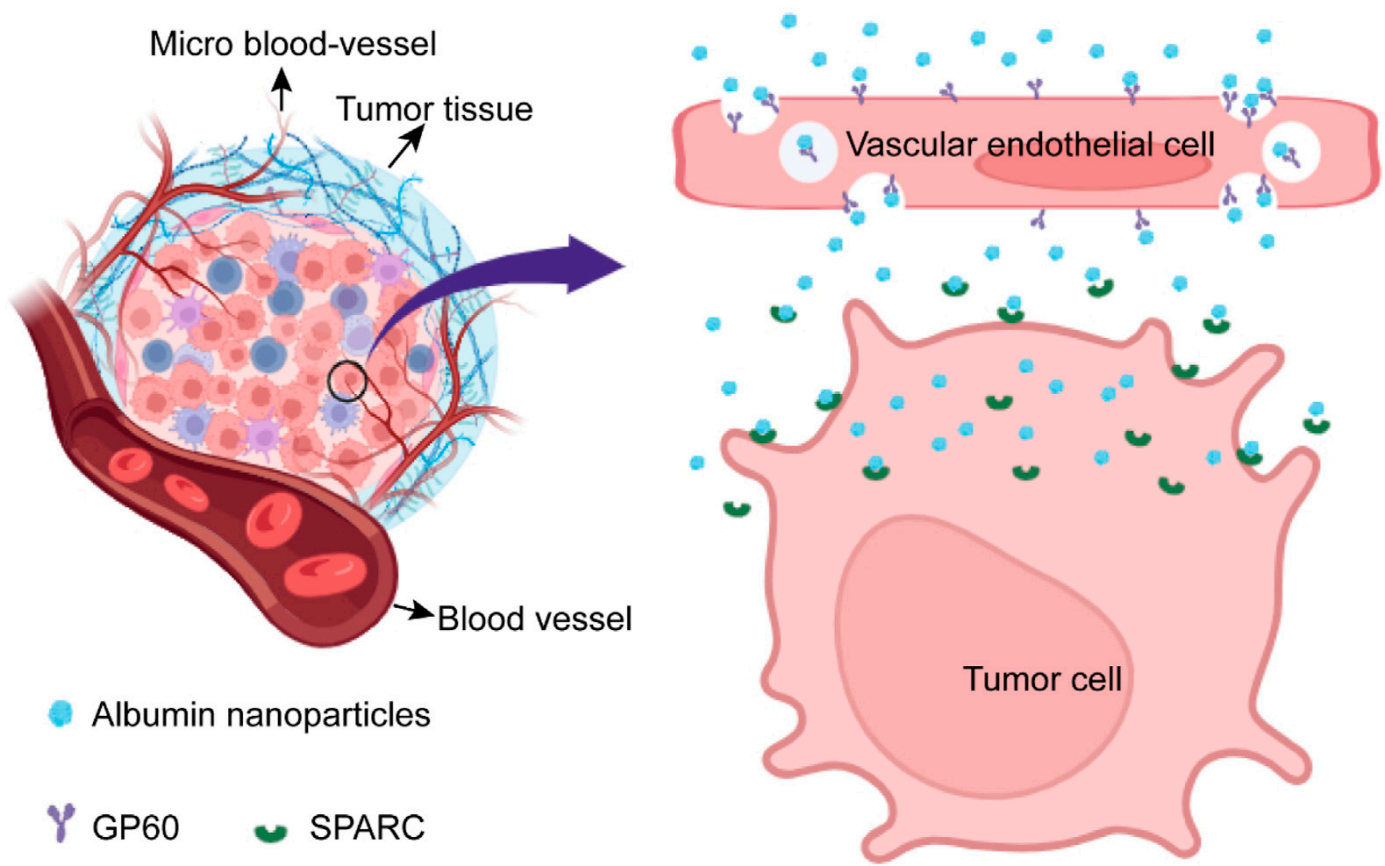
Schematic diagram of albumin nanoparticles targeting tumor cells via the GP60 and SPARC pathways. When albumin nanoparticles are circulated to tumor tissues via intravenous injection, albumin nanoparticles will rely on GP60 receptors to penetrate vascular endothelial cells and enter the tumor microenvironment. Then, these albumin nanoparticles will interact with the SPARC protein secreted by tumor cells, ultimately achieving the effect of targeting tumor cells.

With the development and advancement of science and technology over time, more and more researchers are focusing on drug-targeted delivery, especially the construction of drug carriers with biological activities. Over the past decade, researchers have attempted to construct a number of drug delivery systems with biological activities, such as ferritin nanocarriers, cell membrane-coated drug carriers, and activated protein nanoparticles (Liang et al., 2014; Lee et al., 2016; He et al., 2019; Fang et al., 2023). In this section, protein drug carriers constructed based on the albumin–GP60 interaction and albumin–SPARC interaction are highlighted. The drug delivery system constructed on the basis of albumin first crosses the barrier of vascular endothelial cells into the interstitial space of tumor tissues through the mediation of GP60, and then, drug carriers bind to SPARC expressed by tumor cells, ultimately achieving the goal of targeting tumor cells (Figure 3).

In 2005, Abraxane^®^, constructed based on HSA, first utilized the GP60 and SPARC pathways to enhance the therapeutic effects of paclitaxel. In addition, this drug has achieved more favorable therapeutic results. To this day, Abraxane^®^ still maintains a high market share. Since 2005, more and more studies have begun to try to construct albumin drug carriers to treat various types of tumors. In 2011, Iancu et al. constructed multi-walled carbon nanotubes functionalized with HSA, and their findings demonstrated good targeting effects on HepG2 cells (Lee et al., 2016). In 2016, Lee et al. prepared a kind of albumin nanoparticles that accumulated in the tumor site of an HCT116 cell-xenograft mouse model, and the results demonstrated excellent tumor targetability via a GP60-mediated transcytosis mechanism (Lee et al., 2016). In 2018, a kind of HSA nanoparticles loaded with paclitaxel was constructed for the targeted therapy of glioma by Ruan et al., and this kind of albumin nanoparticles can effectively cross the blood–brain barrier to target brain capillary endothelial cells and U87 cells. All results in the research showed a satisfactory antitumor effect and could serve as a novel strategy for the treatment of glioma (et al., 2018). One year later, a kind of albumin nanoparticles loaded with pirarubicin was constructed to treat the occurrence of cancer and the metastasis of tumors (Zhou et al., 2019). In 2021, Hama et al. investigated evidence for the delivery of Abraxane^®^ via the denatured albumin transport system in detail, with data indicating that Abraxane-derived HSA was taken up into endothelial cells or tumor cells by a mechanism different from normal endogenous albumin. These data thus provided new scientific rationale for the development of a novel albumin drug delivery strategy via a denatured albumin receptor (Hama et al., 2021). Our previous work showed that biologically active albumin nanoparticles can effectively target solid tumors via GP60 and SPARC pathways and can effectively inhibit tumor growth and prolong the survival time of tumor-bearing mice (Meng et al., 2023). Albumin drug delivery systems constructed based on the GP60 or SPARC pathway are numerous. Table 2 summarizes in detail the type of albumin used, the receptor pathway, the type of application, and the level of experimentation.

**TABLE 2:** Study of drug delivery systems constructed on the basis of albumin–GP60 or albumin–SPARC interaction in the last decade.

Types of albumin	Receptor	Drug	Drug-loading method	Types of tumor applications	Cell or animal experiments	References
BSA	GP60 and SPARC	Paclitaxel	Self-assembly	Glioma	Both	Lin et al. (2016)
BSA	GP60 and SPARC	Paclitaxel	Thin-film hydration method	MCF-7 and HepG2	Cell experiments	Chen et al. (2015)
BSA	GP60	Aggregation-induced emission nanoprobes	Embedding method	Glioma	Animal experiments	Gao et al. (2023)
BSA	GP60 and SPARC	Doxorubicin	Self-assembly	Breast cancer	Both	Tan et al. (2021)
HSA	GP60 and SPARC	Paclitaxel	Self-assembly	Prostate cancer	Animal experiments	Li et al. (2014)
BSA	GP60	Gold nanoparticles	Embedding method	Liver cancer	Cell experiments	Mocan et al. (2015)
BSA and HSA	GP60 and SPARC	Doxorubicin	Self-assembly	4T1 breast cancer	Both	Meng et al. (2023)
HSA	GP60 and SPARC	Kolliphor HS 15 and pirarubicin	Thin-film hydration method	B16F10 tumors	Both	Zhou et al. (2019)
HSA	GP60	Doxorubicin	A consequent dropwise mixing and sonication method	HCT116 tumors	Both	Lee et al. (2016)
BSA	GP60 and SPARC	Dipolar oxazepane dye	Chemical cross-linking method	Glioblastoma	Both	An et al. (2021)
HSA	GP60 and SPARC	Doxorubicin	Hydrophobic interaction	4T1 breast cancer	Both	Kang et al. (2023)
BSA	GP60 and SPARC	5-Fluorouracil	Synthesized and covalently coupled method	Breast cancer	Cell experiments	Koziol et al. (2014)
HSA	GP60 and SPARC	Paclitaxel	Self-assembly	Glioblastoma multiforme	Both	Ruan et al. (2018)
HSA	SPARC	Photosensitizer (ZnPcS)	Physical cross-linking method	Malignant gliomas	Both	Li et al. (2023)
BSA	SPARC	Cellax	Physical cross-linking method	EMT6	Cell experiments	Hoang et al. (2015)
HSA	SPARC	Dibenzocyclooctyne	Conjugation	SK-OV3	Both	Park et al. (2020)
BSA	SPARC	Liposomes	Self-modified method	Hepatic fibrosis	Both	Wang et al. (2020)
HSA	SPARC	Paclitaxel	Nab^TM^ technology	Pancreatic cancer	Animal experiments	Kim et al. (2016)
HSA	SPARC	Paclitaxel	Nab^TM^ technology	Pancreatic ductal adenocarcinoma	Animal experiments	Neesse et al. (2014)
HSA	SPARC	Gemcitabine and losartan	Desolvation-cross-linking method	Solid tumor	Animal experiments	Sandha et al. (2023)
HSA	SPARC	Doxorubicin	Physical adsorption	MCF-7 cells	Both	Zhao et al. (2017)
HSA	GP60 and SPARC	Paclitaxel	Self-assembly	MDA-MB-231 human breast cancer	Both	Zhang et al. (2018)
HSA	SPARC	Cisplatin	Conjugation	U87MG glioma	Both	Park et al. (2020)
BSA	SPARC	Albendazole	The desolvation method	Pancreatic carcinoma cells	Cell experiments	Lu et al. (2017)
HSA	SPARC	Trichosanthin	Noncovalent conjugation	Orthotopic breast cancer	Both	Chang et al. (2019)
HSA	SPARC	Paclitaxel	Conjugation	B16F10 melanoma cells	Cell experiments	Park et al. (2017)
BSA	SPARC	Histamine	Conjugation	Multidrug-resistant breast cancer	Both	He et al. (2017)
BSA	SPARC	Lactoferrin	Modification	CT26 peritoneal tumor	Both	He et al. (2022)
HSA	GP60 and SPARC	Exemestane and hesperetin	Hydrophobic interaction	Breast cancer	Both	Gaber et al. (2019)
HSA	SPARC	Docetaxel	Conjugation	SKOV-3 human ovarian cancer cells, B16F10 mouse melanoma cells, NCI/ADR-RES human multidrug-resistant ovarian cells, and 4T1 murine mammary carcinoma cells	Cell experiments	Gad et al. (2018)
HSA	GP60 and SPARC	Paclitaxel	Nab^TM^ technology	Metastatic breast cancer	Human body	Lluch et al. (2014)
HSA	SPARC	Pirarubicin	N/A	4T1 orthotopic mammary tumor	Both	Feng et al. (2022)
HSA	SPARC	Lapatinib	Nab^TM^ technology	Triple-negative breast cancer	Both	Wan et al. (2015)
HSA	SPARC	Temozolomide acid	The desolvation method	Glioma	Both	Helal et al. (2021)
HSA	SPARC	N/A	N/A	Colon cancer	Both	Mi et al. (2017)
HSA	SPARC	Indocyanine green	Noncovalent conjugation	Glioblastoma	Both	Jang et al. (2023)
HSA	SPARC	N/A	N/A	Colorectal cancer	Cell experiments	Zhang et al. (2017)
HSA	SPARC	Eumelanin	Bioconjugation	Breast cancer	Cell experiments	Sanità et al. (2020)
HSA	SPARC	Paclitaxel	Noncovalent conjugation	Pancreatic cancer	Both	Wei et al. (2017)
HSA	SPARC	Silibinin nanocrystals	Embedding method	Liver fibrosis	Both	Luo et al. (2023)
HSA	SPARC	Methotrexate	Hydrophobic interaction	Rheumatoid arthritis	Both	Liu et al. (2019)
BSA	SPARC	(1R,2R3S)-1,2-propanediol acetal-zeylenone	Self-assembly	Canine breast cancer	Animal experiments	Chen et al. (2022)
BSA	SPARC	N/A	N/A	Complex fungal infections	Both	Cheng et al. (2021)
BSA	SPARC	Albendazole	The desolvation method	Ovarian cancer	Both	Noorani et al. (2015)
BSA	SPARC	Formononetin	The inverse solvent precipitation	Lung injury and fibrosis therapy	Both	Ouyang et al. (2023)
HSA	SPARC	Paclitaxel	Nab^TM^ technology	Pediatric sarcomas	Both	Pascual-Pasto et al. (2022)
HSA	SPARC	Paclitaxel	Nab^TM^ technology	Ewing sarcoma	Animal experiments	Pascual-Pasto et al. (2023)
HSA	SPARC	Paclitaxel	Nab^TM^ technology	Pediatric bone sarcoma	Animal experiments	Wagner et al. (2014)
HSA	SPARC	Paclitaxel	Nab^TM^ technology	Pediatric cancer	Human body	Oesterheld et al. (2020)

As shown in Table 2, BSA is significantly more frequently used than HSA, which may be due to several reasons, including the low price of BSA, easy availability of raw materials, and low cost of production (Liu et al., 2022; Zhao et al., 2022). For GP60 receptors and SPARC, SPARC was more frequently used to construct albumin-based drug carriers, which might be due to the reason that SPARC is directly expressed and secreted by tumor cells. Drug carriers constructed based on the albumin–SPARC interaction can reach the tumor tissue or cells more efficiently. Compared to conducting the targeting experiment of GP60 or SPARC at the cellular level only, most research studies about the albumin-based drug delivery system performed both cell experiments and animal experiments, which may be due to the fact that it is more convincing to conduct the targeting experiment of GP60 and SPARC based on the animal experiment level.

In 2016, an article published in *ACS Nano* reported that albumin nanoparticles could penetrate the blood–brain barrier for biomimetic drug delivery. The main principle is to achieve targeted drug delivery through SPARC- and GP60-mediated biomimetic transport. The constructed albumin nanoparticles exhibited enhanced BBB penetration, intra-tumoral infiltration, and cellular uptake, and this research provided a facile method for dual drug-loaded albumin nanoparticle preparation and a promising avenue for biomimetic delivery targeting brain tumors based on combination therapy (Lin et al., 2016). Tan et al. used albumin to prepare chondroitin sulfate-mediated nanoparticles, and the nanoparticles led to greater drug accumulation at the tumor site than with DOX nanoparticles (no albumin modified) or free DOX. The main reason for this effect was that albumin nanoparticles utilized the GP60- and SPARC-mediated pathway for targeted DOX delivery, and the effect resulted in significant inhibition of tumor growth and lower exposure of major organs to DOX (Tan et al., 2021). Li et al. thought nanoparticles using albumin as a particle matrix had entered the mainstream of drug delivery because albumin can interact with its receptors or binding proteins (Li et al., 2014). They concluded that the non-crosslinked formulation was more advantageous for the delivery of paclitaxel by a comparative study. Zhou et al. used the thin-film hydration method to obtain an albumin-bound complex of albumin–pirarubicin (Zhou et al., 2019). In their research, the lack of any chemical reactions preserved albumin bioactivities, and the albumin–pirarubicin complex showed greater tumor accumulation and tumor penetration through GP60- and SPARC-mediated biomimetic transport than pirarubicin and denatured albumin–pirarubicin. An et al. constructed a noble triple-receptor-targeting fluorescent complex based on the GP60- and SPARC-mediated pathway (An et al., 2021). In the study, the imaging of glioblastoma (GBM) cell lines and human clinical GBM tissues was successfully demonstrated, and the study presented great promise for the application of these albumin complexes for GBM identification and surgery at clinical sites. Other research studies of albumin nanoparticles based on the GP60- and SPARC-mediated pathway are shown in Table 2.

Albumin nanoparticles can be enriched in tumor tissue in two ways. The first way is to achieve enrichment in tumor tissue through the receptor protein pathway, and the second way is to achieve enrichment in tumor tissue via an enhanced permeability and retention effect (EPR effect). Why do some studies achieve targeted drug delivery using just one receptor? First, this is related to the choice of enrichment. The use of both GP60 and SPARC receptors together places greater emphasis on targeted drug delivery through the receptor protein pathway. While just one receptor was used, the study placed more emphasis on achieving targeted drug delivery through the combined use of both modalities (the receptor protein pathway and EPR effect). Second, the different delivery modes of albumin nanoparticles also determine whether they can select a single receptor for targeted delivery. For example, delivery by *in situ* injection at the tumor site allows the selection of only the SPARC receptor for targeted drug delivery. If the albumin nanoparticles are administered via intravenous injection, it may be necessary to utilize both GP60 and SPARC receptors to achieve a targeted delivery effect. Third, experimental studies of albumin nanoparticles carried out solely at the cellular experimental level can also achieve targeting effects using only a single receptor.

In 2009, Knauer et al. revealed that the albumin-binding domain of SPARC was located at its C-terminus (Knauer et al., 2009). However, there are no articles on the binding regions of albumin to GP60 and SPARC. In 2017, Mi et al. showed interactions between HSA and SPARC via molecular dynamic simulation; however, the result is also only a visualization of the picture and still does not guide the specific domain of interaction (Mi et al., 2017). In our ongoing research, we tentatively found that the IIAIIB region of albumin may be its binding region to GP60 and SPARC, but this still needs to be supported by more detailed research data.

## 5 Discussion

The denaturation of albumin affects its ability to bind to the GP60 receptor and SPARC, so maintaining the activity of albumin as much as possible helps to strengthen its interaction with the GP60 receptor and SPARC. However, albumin-based drug carriers are often denatured by exposure to organic solvents during preparation, which reduces their ability to bind to the GP60 receptor or SPARC and can even lead to the loss of their ability to bind to the GP60 receptor or SPARC. In order to overcome the problem, strategies on how to make albumin self-assemble into nanoparticles under conditions of invariance must be proposed as soon as possible. We proposed a strategy to make albumin self-assemble into nanoparticles without the addition of organic reagents using the technology of reverse QTY code several months ago. The experimental results showed that albumin nanoparticles modified by the technology of reverse QTY code can maintain its biological activities and target tumor tissue or cells efficiently via the GP60 receptor and SPARC pathway (Meng et al., 2023). Next, we will continue to conduct research to show better effects of tumor treatment in pigs, monkeys, and even human applications as well.

In recent years, a number of studies have demonstrated the importance of maintaining albumin activities for the targeting delivery of albumin-based drug carriers. In 2022, Nisar et al. investigated the interaction and structural modifications of native albumin (BSA) with iron oxide nanoparticles, and the results provided an understanding of the interaction and structural modifications of native albumin (BSA), which has the potential to provide fundamental repercussions in future studies (Nisar et al., 2022). In 2018, Hyun et al. used native albumin to modify polymer nanoparticles for enhancing drug delivery to solid tumors (Hyun et al., 2018). The results showed a surface layer formed with native albumin facilitated nanoparticle transport and drug delivery into tumors via the interaction with albumin-binding proteins, and the study demonstrated that native albumin can enhance the penetration of nanoparticles through the binding receptor or protein pathway. Back in 2016, Miranda et al. investigated the influence of albumin structure and gold speciation on the synthesis of gold nanoparticles (Miranda É et al., 2016). Their results presented that the denaturation of albumin would expose hydrophobic groups to the solvent, and the result would weaken the ability of the albumin–receptor interaction. This research also confirmed maintaining the biological activities of albumin as key for the targeting of albumin-based carriers. In addition, Wu et al. found albumin with biological activities could enhance the transport of copolymer nanoparticles, and this study confirmed similarly that maintaining the biological activities of albumin is important for targeting the delivery of albumin-based carriers (Wu et al., 2013).

Native albumin has numerous drug-binding sites. If researchers do not need to self-assemble albumin into nanoparticles but just use a single molecule of albumin for drug delivery, it will reduce the chance of denaturing albumin because there is no need to add organic solvents to induce albumin to self-assemble into nanoparticles. Thus, the use of single-molecule albumin to load antitumor drugs may be an effective way to strengthen the efficiency of the albumin–GP60 and albumin–SPARC interactions. In addition, if albumin can be analyzed by rational bioinformatics methods and modified to show amphiphilicity, then the amphiphilic albumin obtained from the modification can also self-assemble into nanoparticles without the addition of chemically induced reagents, and thus, its biological activities can be well preserved.

## 6 Conclusion

GP60 is overexpressed in vascular endothelial cells, and SPARC is overexpressed in most tumor cells, such as breast cancer, glioma, melanoma, and liver cancer. GP60 and SPARC act as receptor proteins of albumin, so they can interact with albumin closely. Therefore, GP60 and SPARC can be key targets for tumor therapy. Albumin drug delivery systems constructed on the basis of the albumin–GP60 interaction and albumin–SPARC interaction can target tumor tissue or cells via GP60 and SPARC pathways. However, denatured albumin has a diminished ability to bind to GP60 or SPARC, so how to maximize the preservation of albumin activity is the key to the effective use of GP60 or SPARC for cancer therapy.
